# Anxiety and Depression among Patients with Coronary Artery Disease Attending at a Cardiac Center, Kathmandu, Nepal

**DOI:** 10.1155/2018/4181952

**Published:** 2018-11-25

**Authors:** Puja Sharma Dhital, Kalpana Sharma, Pratik Poudel, Pankaj Raj Dhital

**Affiliations:** ^1^Advanced Adult Nursing, Nepal Polytechnic Institute, Bharatpur, Nepal; ^2^Advanced Adult Nursing, Chitwan Medical College, Bharatpur, Nepal; ^3^Department of Radiology, College of Medical Sciences, Bharatpur, Nepal; ^4^Department of Agricultural Extension and Rural Sociology, Agriculture and Forestry University, Rampur, Nepal

## Abstract

Psychiatric morbidity such as anxiety and depression is common among patients with coronary artery disease (CAD). The coexistence of psychiatric morbidity negatively affects the outcome of treatment and increases the overall burden of disease in CAD patients. The objective of the study was to identify the level of anxiety and depression among patients with CAD. Descriptive, cross-sectional research design and purposive sampling were used and a total of 168 patients having coronary artery disease were selected purposively for the study from the patients attending cardiac outpatient department of Sahid Gangalaal National Heart Center, Kathmandu, Nepal. Data was collected on 2017 by using pretested semistructured interview schedule, Hospital Anxiety and Depression Scale. The findings showed that 27.4% of the respondents had anxiety caseness and 23.8% of the respondents had depression caseness. Bivariate analysis showed that patient's level of anxiety was significantly associated with sex, family income, occupation status, and self-esteem. Higher level of anxiety (42.4%) was found in female than male patients. Likewise, level of depression was significantly associated with education status, occupation status, presence of comorbidities, physical exercise, and self-esteem of the patients. There was significant positive relationship between anxiety and depression score. Thus anxiety and depression were common among patients with CAD. Hence, anxiety and depression in CAD patients need be monitored regularly, provide regular counseling services, and refer the patients for the treatment when needed.

## 1. Introduction

Noncommunicable diseases (NCDs) are the common public health problem worldwide. They cause 70% of deaths globally. Almost three quarters of all NCD deaths and 82% of the 16 million people who died prematurely, or before reaching 70 years of age, occur in low- and middle-income countries [1]. Among all NCDs, cardiovascular disease (CVD) is the leading global cause of death globally [2]. Nepal is also facing the surging burden of NCDs where the prevalence of NCDs is 31%. Among NCDs, CVD accounts 40% of all NCDs cases. Majority of CVD cases were hypertension (47%) followed by cerebrovascular accident (16%), congestive cardiac failure (11%), ischemic heart disease (7%), rheumatic heart disease (5%), and myocardial infarction (2%) [3].

Psychiatric morbidity such as depression and anxiety are common among patients with CHD. One study indicated that 32.5% and 17.5% of patients with CHD have depression and anxiety symptoms, respectively [4]. Most studies showed depression as an important disorder that leads to an increase in cardiovascular events, readmission to hospital, and CHD mortality [5]. The coexistence of physical and psychiatric morbidity negatively affects the course and outcome of both the conditions resulting in increased overall burden of disease [6].

Identification of psychiatric disorders (anxiety and depression) in CAD patients has shown to improve prognosis and quality of life of patients with CAD [7]. Patients treated for their depression and anxiety might better adhere to risk factor modifications, prescribed medications, and rehabilitation programs. Therefore, patients with known CAD with evidence of psychiatric morbidity should be evaluated [8]. Around 95.4% of patients with ischemic heart disease (IHD) reported psychiatric symptoms, either depression or anxiety. Major depressive disorder was found in 34.6% patients and anxiety disorder was present in 36.9% patients. Majority of patients with poor quality of life were in the domain of anxiety/depression [9]. Similarly, anxiety and depression were present in 48.5% and 25.2% of the patients with myocardial infarction (MI) [10].

High or increasing level of anxiety that is maintained over an extended period is associated with an increased risk of MI and death in patients with CAD [11]. Depressed individuals are less likely to practice healthy habits, so they generally have more of these risk factors compared to those without depression. Nonadherence, which includes the improper use of drugs, not following a prescribed diet or exercise programme, or not visiting doctor on scheduled appointment, may be behavioral risks that may lead to the development and worsening of CAD. Depression has been shown to be a risk factor for poor medication adherence and cardiovascular outcomes with poor adherence have worst prognosis [8]. Various factors are associated with anxiety and depression of patients with CAD. A study in a tertiary hospital of Malaysia among 108 CHD patients revealed that patients with CHD had a low level anxiety and depression scores. Unmarried respondents with comorbid disease have higher anxiety and depression than married and respondents without comorbid disease [12]

The general objective of the study was to find out anxiety and depression among patients with coronary artery disease.

## 2. Methodology

Descriptive cross-sectional research study design was used to find out the anxiety and depression of patients with CAD attending Shahid Gangalal National Heart Center (SGNHC), Bansbari, Kathmandu, Nepal. The nonprobability, purposive sampling technique was used to select the required sample size. Researcher identified the sample from OPD by verbally asking the patients about their age and purpose of visit to OPD. Then medical file was reviewed to confirm the information given by patient. Semistructured interview schedule for the sociodemographic variables, disease related variables, behaviour related variables, and support system was developed by researcher based on extensive literature review. Hospital Anxiety and Depression Scale (HADS), developed by Zigmond and Snaith in 1983 and validated among Nepalese people by Risal et al. on 2015, was used to assess anxiety and depression. It had 7 items related to anxiety and 7 items related to depression [13]

## 3. Statistical Analysis

The data was edited, coded, and entered in EpiData3.1 and then exported to IBM SPSS 20 program for analysis. Data was analyzed using descriptive statistics, i.e., frequency, percentage, mean, and standard deviation to describe the patient's demographic variables, anxiety, and depression. Chi square test was used to determine association between different selected variables with level of anxiety and level of depression. Spearman's correlation coefficient test was used to find out the relationship between anxiety and depression of the patients with CAD.

## 4. Results

Table 1 shows that, out of 168 respondents, 57.7% were between the ages of 40-60 years. The mean age of the respondents was 53.01±13.91 years. Similarly, 60.7% were male, 96.4% were married, and 80.3% were living with their spouse.

Table 2 shows that two-thirds (66.1%) of the respondents were diagnosed with myocardial infraction followed by angina pectoris (20.2%) and ischemic heart failure (13.7%). Regarding mode of treatment, more than two-thirds (69.1%) of the respondents had surgery. Likewise, half of the respondents (50.0%) had other comorbid conditions.

Table 3 shows that 27.4% of the respondents had anxiety caseness and 19.6% had borderline anxiety. Similarly, 26.2% of the respondents had borderline depression and 23.8% had depression caseness.

Table 4 shows that the level of anxiety was significantly associated with sex of the respondents where females had more anxiety cases than males. Moreover, respondents who were living single had more anxiety caseness than respondents who were living with their spouse. Similarly, respondents who were involved in housework had more anxiety caseness than other occupation. The results further demonstrated that respondents whose family income is not sufficient to family had more anxiety caseness than respondents whose family income is not enough to run family.

Table 5 shows that respondents who were living single had more depression caseness than respondents who were living with their spouse. Likewise, level of depression was more prevalent among illiterate respondents having CAD than literate respondents having CAD, which further demonstrate that the higher the education the lower the depression cases. Moreover, respondents who were involved in agriculture had more depression caseness than other occupation.

Table 6 shows that there was significantly positive correlation between anxiety and depression (0.482).

## 5. Discussion

Out of 168 patients, 27.4% of the patients had anxiety caseness and 19.6% had borderline anxiety. This finding is almost similar to the study conducted in Brazil [14] where 48.4% of CAD patients had anxiety. Similarly, studies conducted in Brazil [4] and Germany [15] showed 32.5% and 8.3% of anxiety among CAD patients, respectively. Anxiety among CAD patients is higher in the present study which might be due to unemployment status after illness, illiteracy, lack of awareness regarding prognosis of CAD, and limited counseling facility in the healthcare setting.

In this study, 23.8% patients had depression caseness and 23.8% and 26.2% had borderline depression, whereas studies conducted in Brazil [14] and Germany [15] showed that 26.4% and 5.9% of CAD patients had depression, respectively. Depression in CAD patients is higher in this study which might be due to lack of awareness and limited accessibility and availability of health services facility including health insurance.

In this study, sex of the patients was significantly associated with level of anxiety of the CAD patients where females had higher level of anxiety than males. This finding is supported by the studies conducted in Brazil [4] and America [11] which showed the higher level of anxiety in female CAD patients. Females are more prone to have anxiety which might be due to their multiple roles, gender discrimination, or other family problems. Living status was another significant variable which influence the level of anxiety of CAD patients where patients who were living alone had higher level of anxiety than the patients living with spouse. This finding is consistent with the study conducted in India [16] which revealed that patients who were living alone had more anxiety. This might be due to lack of physical, emotional, and financial support among those CAD patients who were living alone.

Moreover, family income and occupation status were also associated with level of anxiety of CAD patients where patients whose annual family income was not sufficient to run their family had higher anxiety level. However study conducted in Pakistan [17] showed that there was no significant relation of anxiety with socioeconomic and occupation status of CAD patients. The discrepancy in findings might be due to variation in sample size and sample characteristics. This study found that the patients who had higher level of self-esteem had lower level of anxiety compared to patients who had lower level of self-esteem (p ≤ 0.001). In contrast to this finding, the study conducted in Brazil [4] revealed that the patients who had higher self-esteem score had higher level of anxiety. This discrepancy in the finding of the studies might be due to inclusion of different nature of sample and health service facilities which helps to maintain their higher self-esteem.

The findings of the study showed that level of anxiety was not associated with age and comorbid conditions of the CAD patients. This finding contradicts with the finding of the study conducted in United States [11] where age was significantly associated with level of anxiety. Likewise, study conducted in India [16] showed that level of anxiety of CAD patients had significant association with comorbid condition. The discrepancy in findings might be due to variation in sample size, study setting, and characteristics of sample.

The finding of the study showed that people who were living alone had greater level of depression (p≤0.001) than the patients living with their spouse. This finding is similar to the study conducted in Malaysia [12] where patients living alone had more depression than the patients living with their spouse. This might be due to lack of ultimate care and support of family members which is pivotal to the individual who are diseased. Likewise, patients who had higher level of education had lower level of depression (p=0.017). In contrast to this finding, study conducted in Malaysia [12] revealed the education level to be not associated with depression. This discrepancy in findings might be due to inclusion of different nature of samples and study setting of the study.

Similarly, occupation was identified as one of the influencing variable for the level of depression of CAD patients (p=0.001) in which patients who were involved in housework had higher level of depression compared to patients involved in others occupations (agriculture, service, and business). In contrast to this finding, the study conducted in Pakistan [17] revealed that occupation status was not associated with level of depression. This might be due to use of different tool or inclusion of different nature of sample population. The finding of the study revealed that the comorbidities were significantly associated with the level of depression of CAD patients and this finding is consistent with the study conducted in Malaysia [12]. This might be due to more symptoms related to the associated diseases which interrupt the daily activities of the CAD patients which caused them to feel more depressed. Moreover, physical exercise was significantly associated with the level of depression (p=0.001) of the CAD patients where the patients who performed regular exercise had low level of depression. This might be due to role of exercise in reduction of stress in the individual and enhancement of the overall well-being.

Further, this study revealed that there was significant association between self-esteem and level of depression of the CAD patients where patients with higher level of self-esteem had lower level of depression. This might be due to association of positive self-esteem with mental well-being, happiness, adjustment, success, achievements, and satisfaction where low self-esteem can contribute to negative outcomes such as depression. In contrast to this finding, the finding of study conducted in Brazil [4] revealed that the patients who had higher self-esteem score had higher level of depression. This discrepancy in the finding of the study might be due to inclusion of different group of sample population in the studies. In this study level of depression was not significantly associated with sex of the CAD patients, whereas study conducted in Brazil [4] showed that depression had significant association with sex of the patients. The discrepancy in findings might be due to variation in sample population and study setting.

## 6. Conclusion

In conclusion, coronary artery disease and depression are both highly prevalent diseases. Both of them cause a significant decrease in quality of life for the patient and impose a significant economic burden on society. Anxiety and depression have great correlation in CAD patients. So psychiatry visits by specialties along with assessment by nurses in cardiovascular patients are recommended for case finding in anxiety and depression.

## 7. Recommendation

Nurse Manager should facilitate the introduction of health teaching for CAD patients and their family members in order to inform them about positive prognosis after better treatment and management which helps to lower the anxiety and depression. The different methods of psychological adaptation for anxiety and depression and CAD related effects should be developed based on best available evidence and the findings of this study. The design of the psychological adaptation should consider the involvement of the family/caregivers. This intervention should be piloted and tested using an experimental study. Similar study can be conducted on multicenter setting covering larger population.

## Figures and Tables

**Table 1:** Sociodemographic variables of the respondents n= 168.

**Socio-demographic Variables**	**Frequency**	**Percentage**
**Age group (in years)**		
19-39	33	19.6
40-64	97	57.8
65 above	38	22.6
*Mean ± SD=53.01±13.91 Min:20 Max:79*		
**Sex **		
Male	102	60.7
Female	66	39.3
**Living with**		
Spouse	135	80.4
Single	33	19.6
**Type of family **		
Nuclear	85	50.6
Joint	83	49.4
**Educational status**		
Literate	104	61.9
Illiterate	64	38.1
**Employment status after illness**		
Employed	106	63.1
Unemployed	62	36.9
**If employed, occupation(n= 106)**		
Housework	32	30.2
Agriculture	22	20.7
Service	27	25.5
Business	25	23.6

**Table 2:** Disease related variables of the respondents n= 168.

**Variables**	**Frequency**	**Percent**
**Type of CAD**		
Angina pectoris	34	20.2
Myocardial infraction	111	66.1
Ischemic heart failure	23	13.7
**Mode of treatment**		
Medical	52	30.9
Surgical	116	69.1
**Presence of co-morbidities**		
Yes	84	50.0
No	84	50.0
**Family history of CAD**		
Yes	20	11.9
No	148	88.1

**Table 3:** Level of anxiety and depression of the respondents n= 168.

**Level **	**Frequency**	**Percentage**
**Anxiety**		
No anxiety (0-7)	89	53.0
Borderline anxiety (8-10)	33	19.6
Anxiety caseness (11-21)	46	27.4
**Depression**		
No depression (0-7)	84	50.0
Borderline depression (8-10)	44	26.2
Depression caseness (11-21)	40	23.8

Total	168	100.0

*Possible score of anxiety: 0-21; possible score of depression: 0-21*

**Table 4:** Association of level of anxiety with different variables.

**Variables**	**Level of Anxiety**			χ**2**	**p-value**
	**No anxiety n (**%**)**	**Borderline anxiety ** **n (**%**)**	**Anxiety caseness ** **n(**%**)**		
**Gender**					
Male	66(64.7)	18(17.6)	18(17.6)	16.254	<0.001
Female	23(34.8)	15(22.7)	28(42.4)		
**Living with**					
Spouse	76(56.3)	28(20.7)	31(23.0)	6.751	0.034
Single	13(39.4)	5(15.2)	15(45.5)		
**Occupation status (n= 106)**					
Housework	8(25.0)	8(25.0)	16(50.0)	21.995	0.001^*∗*^
Agriculture	8(36.4)	6(27.3)	8(36.4)		
Service	16(59.3)	6(22.2)	5(18.5)		
Business	20(80.0)	2(8.0)	3(12.0)		
**Economic status**					
Enough to run family	78(62.4)	21(16.8)	26(20.8)	17.921	<0.001
Not enough to run family	11(25.6)	12(27.9)	20(46.5)		

*Significance level at 0.05. ∗Likelihood ratio.*

**Table 5:** Association of level of depression with different variables.

**Variables **	**Level of depression**			χ**2**	**p-value**
	**No depression** **n(**%**)**	**Borderline depression ** **n (**%**)**	**Depression caseness n(**%		
**Living with**					
Spouse	76(56.3)	36(26.7)	23(17.0)	18.748	<0.001
Single	8(24.2)	8(24.2)	17(51.5)		
**Education status**					
Literate	64(61.5)	25(24.0)	15(14.4)	17.854	<0.001
Iliterate	20(31.2)	19(29.7)	25(39.1)		
**Level of education **					
Up to secondary	16(43.2)	13(35.1)	8(21.6)	8.129	0.017
Above secondary	48(71.6)	12(17.9)	7(10.4)		
**Occupation status (n= 106)**					
Housework	11(34.4)	10(31.2)	11(34.4)	23.616	0.001*∗*
Agriculture	6(27.3)	7(31.8)	9(40.9)		
Service	21(77.8)	4(14.8)	2(7.4)		
Business	18(72.0)	5(20.0)	2(8.0)		
**Presence of co morbidities**				7.233	0.027
Yes	35(41.7)	22(26.2)	27(32.1)		
No	49(58.3)	22(26.2)	13(15.5)		
**Family history of CAD**				0.482	0.786^*∗*^
Yes	11(55.0)	4(20.0)	5(25.0)		
No	73(49.3)	40(27.0)	35(23.6)		

*Significance level at 0.05. ∗Likelihood ratio.*

**Table 6:** Relationship between anxiety and depression score of the respondents n= 168.

**Variables **	**Anxiety**	**Depression**
Anxiety	1	-
Depression	*∗∗*0.482	1

*P value< 0.001. ∗∗Correlation is significant at 0.05 level (2 tailed).*

## Data Availability

The data used to support the findings of this study are available from the corresponding author upon request.
